# In vivo assessment of prostate cancer response using quantitative ultrasound characterization of ultrasonic scattering properties

**DOI:** 10.1186/s12885-021-08706-7

**Published:** 2021-09-03

**Authors:** Deepa Sharma, Laurentius Oscar Osapoetra, Mateusz Faltyn, Anoja Giles, Martin Stanisz, Gregory J. Czarnota

**Affiliations:** 1 Physical Sciences, Sunnybrook Research Institute, Toronto, ON Canada; 2 Department of Radiation Oncology, Sunnybrook Health Sciences Centre, Toronto, ON Canada; 3 Department of Radiation Oncology, University of Toronto, Toronto, ON Canada; 4 Department of Medical Biophysics, University of Toronto, Toronto, ON Canada

**Keywords:** Cell death, Hyperthermia, Prostate cancer, Quantitative ultrasound spectroscopy

## Abstract

**Background:**

The study here investigated quantitative ultrasound (QUS) parameters to assess tumour response to ultrasound-stimulated microbubbles (USMB) and hyperthermia (HT) treatment in vivo*.* Mice bearing prostate cancer xenografts were exposed to various treatment conditions including 1% (v/v) Definity microbubbles stimulated at ultrasound pressures 246 kPa and 570 kPa and HT duration of 0, 10, 40, and 50 min. Ultrasound radiofrequency (RF) data were collected using an ultrasound transducer with a central frequency of 25 MHz. QUS parameters based on form factor models were used as potential biomarkers of cell death in prostate cancer xenografts.

**Results:**

The average acoustic concentration (AAC) parameter from spherical gaussian and the fluid-filled spherical models were the most efficient imaging biomarker of cell death. Statistical significant increases of AAC were found in the combined treatment groups: 246 kPa + 40 min, 246 kPa + 50 min, and 570 kPa + 50 min, in comparison with control tumours (0 kPa + 0 min). Changes in AAC correlates strongly (*r*^*2*^ = 0.62) with cell death fraction quantified from the histopathological analysis.

**Conclusion:**

Scattering property estimates from spherical gaussian and fluid-filled spherical models are useful imaging biomarkers for assessing tumour response to treatment. Our observation of changes in AAC from high ultrasound frequencies was consistent with previous findings where parameters related to the backscatter intensity (AAC) increased with cell death.

**Supplementary Information:**

The online version contains supplementary material available at 10.1186/s12885-021-08706-7.

## Introduction

Treatment response monitoring is crucial in the management of cancer progression. Depending on the severity of cancer and its type, a treatment plan can be adjusted if needed. One of the most commonly performed tests to diagnose and examine treatment response is biopsy. This involves invasive procedures to access tissue or collect cell samples [1] and is associated with inherent risks. Additionally, the involvement of several imaging technologies such as ultrasound (US), magnetic resonance imaging (MRI), and computed tomography (CT) for guidance can turn these into expensive procedures [2–5]. Several non-invasive alternatives to biopsy for response monitoring have been developed and studied, one of which includes a quantitative ultrasound (QUS) spectroscopy.

QUS spectroscopy is a non-invasive, versatile, relatively inexpensive, and novel imaging modality that demonstrates broad applications within medicine. It is widely used for facile and rapid assessment of therapy response by distinguishing dying tumour cells from viable ones [6]. QUS spectroscopy analyzes frequency-dependent power spectrum or backscatter coefficient from US radiofrequency (RF) data [7, 8]. This analysis results in spectral parameters that can be used to distinguish between tissue types and states [9]. Several parameters including the mid-band fit (MBF), 0-MHz intercept (SI) and, spectral slope (SS) can be readily determined from US-RF data and hence can be used for characterizing tissue structural changes based on acoustic properties contrast. These acoustic parameters are linked to microscopic and macroscopic tissue properties. The MBF is known to be associated with the size, concentration, and attenuation of scatterers whereas SI and SS are measures of acoustic scatterer concentration and size, and the size of acoustic scatterers, respectively [10, 11]. Several findings have now confirmed that changes in these spectral parameters are directly correlated with alteration in tissue morphological structures [12–14]. Specifically, studies have demonstrated that cells undergoing apoptosis exhibited changes in spectral parameters compared to normal cells [13, 14]. Furthermore, these outcomes were generally consistent using either conventional-frequency (1–10 MHz) or the high-frequency US (> 20 MHz) [14].

Several other US backscatter parameters, such as the average scatterer diameter (ASD), and the average acoustic concentration (AAC) can also be estimated using US-RF data. Both of these parameters are known to be useful and important indicators for monitoring tumour response and differentiating tumour types. More specifically, patients diagnosed with breast cancer that responded to chemotherapy displayed a substantial escalation in AAC parameter as early as week one with a maximum increase at week 8. On the contrary, non-responder patients demonstrated no such changes following treatment [15]. Tadayyon et al. also reported similar results from preclinical models using breast tumour (MDA-MB-231) xenografts followed by chemotherapy. Their study indicated a strong correlation between the AAC parameter and cell death across tumour sections after 24 h. A significant increase in AAC with an increase in microstructural changes was reported [16].

In this study, mice bearing PC3 prostate-xenograft tumours were exposed to US-mediated microbubble (MB) perturbations. Animals were exposed to 16 cycles of tone burst at 500 kHz center frequency and 3 kHz pulse repetition frequency for 5 min within sonification over 750 milliseconds spread over this period. Peak negative pressure was varied from 0 kPa, 246 kPa, or 570 kPa, corresponding to a mechanical index (MI) of 0.18, 0.35, and 0.80, at the focus. Prior to US exposure, Definity microbubbles were administered based on our previous research (1% (v/v)) [17–20]. Five hours after MB-stimulation, animal tumours were exposed to water bath hyperthermia at 43 °C; in a water bath for 0, 10, 40, and 50 min (detail of treatment conditions and permutations was previously described elsewhere [19]. We estimated and examined the US backscatter parameter-AAC of prostate cancer (PC3) xenografts before and 24 h after exposure to USMB and HT treatment. Results obtained were further compared with tissue morphological changes and cell death using histopathological analysis. The study supports previous findings which have demonstrated that cell-death-inducing cancer therapy induces a greater increase in tissue microstructural changes that leads to an increase in AAC [16]. In this study, a novel combination of USMB and HT resulted in the enhancement of overall cell death. QUS spectroscopy using high US frequencies was used to evaluate tumour response to therapy. Scatterer property estimate in AAC was found to be a useful imaging biomarker of cell death. Changes in AAC strongly correlated with cell death. Significant increases in AAC were observed in the combined treated groups: 246 kPa + 40 min, 246 kPa + 50 min, 570 kPa + 40 min, and 570 kPa + 50 min. Histopathological analysis confirmed positive staining of cell death in these treatment groups, showing morphological characteristics of apoptosis and necrosis. This study demonstrates the potential of QUS spectroscopy for the non-invasive evaluation of tumour response to cancer treatment.

## Materials and methods

### Cell culture

Human prostate cancer cells (PC3) obtained from American Type Culture Collection (ATCC, Manassas VA, USA) were cultured in RPMI-1640 (Wisent Inc., St Bruno, Canada) media supplemented with 5% penicillin/streptomycin antibiotic (Sigma-Aldrich) and 10% fetal bovine serum (Sigma-Aldrich, St Louis, MO, USA). Cells were exposed to 5% CO2 HEPA-filtered air and were incubated at 37 °C. Confluent cells were then harvested at room temperature with 0.02% EDTA and 0.25% trypsin solutions. Cell pellets were isolated and centrifuged at 4 °C for 10 min before being re-suspended in Mg+/Ca + Dulbecco’s Phosphate Buffered Saline (DBPS).

### Animal model

Five to six-week-old CB-17 severely combined immunodeficiency (SCID) male mice obtained from Charles River Inc. (Wilmington, MA, USA) were used for this study. A total volume of 5 × 10^6^ PC3 cell suspension was injected subcutaneously into the lower right hind leg of each animal. Tumours were grown for approximately 2–3 weeks to reach a maximum diameter of 7–10 mm prior to experiments.

Mice were anesthetized via intraperitoneal injection with a mixture of ketamine (100 mg/kg body weight), xylazine (5 mg/kg body weight), and acepromazine (1 mg/kg body weight) (Sigma, Burlington, ON, Canada), and 2% oxygen ventilated isoflurane. To ensure optimal body temperature of the mice, both heating lamps and heating pads were utilized during experiments to keep the mice warm. All animals were closely monitored for irregular breathing, and oxygen was administered to animals appearing to be in distress.

### Ethics statement

All animal experimental procedures were conducted in compliance with the guidelines from Canadian Council on Animal Care. Experimental protocols were approved by the Sunnybrook Health Science Centre Institutional Animal Care and Use Committee. Animals were visually monitored and handled with great care to minimize suffering or pain throughout all experimental procedures. All animals used in the study were euthanized at the end of the study either by cervical dislocation (deemed as standard operating procedures by comparative research) followed by anesthetic overdose.

### Experimental design

Tumour response to HT and USMB treatments was assessed after 24 h. Treatments included HT only (10, 40, and 50 min), microbubbles only, and a combination of USMB and HT treatments. 3–5 mice were used for each treatment condition.

### Ultrasound and microbubble treatment

Definity microbubbles (1% (v/v)) (perfluropropane gas/liposome shell, Lantheus Medical Imaging, Inc., North Billerica, MA, USA) stimulated at 246 kPa and 570 kPa US pressures were used for this study. Microbubbles were created and activated using a Vial mix (Lantheus Medical Imaging, North Billerica, MA, USA) device at 3000 rpm for 45 s prior to animal injection. Microbubble concentrations were calculated according to total animal blood volume estimated by animal weight. Mice to be treated with microbubbles and microbubble/HT combination treatments were fitted with a tail vein catheter to allow for injection of saline and microbubbles. Following the microbubble injection, mice were given a second injection of 0.1 ml saline to flush the tail vein catheter.

The US therapy system consisted of a digital acquisition system (Acquiris CC103, Agiulent Technologies NY), an amplifier (RPR4000, Ritec Inc.), a waveform generator (AWG530, Tektronix), and a transducer with 500 kHz frequency and 28.6 mm aperture diameter. During USMB treatments, each mouse was mounted on a custom stage which was partially immersed in a 37 °C water bath in such a way that placed the tumour at the natural peak focus of the US transducer. The tumour bearing hind leg of each animal was exposed to US immediately following MB injection within the half-maximum peak of the acoustic signal. Animals were exposed to a 16 cycle tone burst at 500 kHz with a 3 kHz pulse repetition frequency, resulting in an average duty cycle of 0.25% for a total of 5 min. Every 2 s, acoustic bursts were repeated to allow blood vessels to refill with MB between insonification, causing bubble bursting in vivo.

### Hyperthermia treatment

Animals were administered HT treatments 5 h following USMB treatments. Animals were mounted in 50 ml custom-made falcon tubes consisting of a hole at the bottom to act as an air vent, a hole in the opening lid for the leg and tail to go through, and a strip of Velcro. The tail and treatment leg of each mouse was fixed using tape and attached to the customized plate with corresponding Velcro attachments. The plate was immersed into a 43 °C water bath, held in place by a three-prong extension clamp attached to a support stand. The tumour bearing leg of each animal was then submerged into the water for their respective treatment times of 10, 40, or 50 min.

### Data acquisition and analysis

 US-RF and B-mode data were collected 24 h before and after all treatments using a VEVO 770 imaging system (VisualSonics Inc., Toronto, Canada). The system was equipped with a single element RMV-710B transducer. The reported characteristics of the high frequency transducer are: 25 MHz center frequency, 15 mm focal depth, and f/2.1 [21, 22]. The measured center frequency was 20 MHz, providing 10 MHz to 25 MHz -6 dB bandwidth. The measured axial and lateral resolutions were 54 μm and 149 μm, respectively. For volumetric RF data acquisition, we used the following acquisition settings: 0.2 mm step size and 20 dB gain. The step size is the elevation (out-of-plane) distance between successive volumetric RF frames, while the gain is the receiver amplifier gain for the RF signal.

### Backscatter parameter estimations

We selected ten different regions of interest (ROIs) from the tumour for spectral analysis. These ROIs correspond to 10 imaging planes within the tumour volume. ROIs were selected in the center of the tumour, accounting for approximately 2/3 of the tumour cross-sectional area (approximately 5–10 × 5–10 mm in-plane and 5–10 mm through-plane). QUS spectral parameters were estimated from each frame (10 frames per animal/tumour) and subsequently averaged across the US scan.

We used the sliding window technique to obtain QUS spectral parametric images that reflect the spatial distribution of acoustic properties within the tumour. These properties have been shown to be potential imaging biomarkers of cell death as the tumour responds to various modes of cancer therapy. We utilized a 10λ by 10λ sliding window with 80% overlap between adjacent windows in both the axial and the lateral directions. Here, λ is the wavelength of the acoustic waves. The size of the window was chosen to include enough scatterers for reliable spectral estimation while preserving image texture. A Hanning gating function was applied on individual RF scan lines within the window. Subsequently, the power spectrum of the gated RF signals within the window was estimated using the fast Fourier transform (FFT) method. Averaged power spectrum within the window was estimated for effective spectral parameter estimation. In order to remove US system-dependent effects, normalized power spectrum (NPS) was estimated using a reference phantom technique [23]. The reference phantom consisted of glass beads of 3.3 ± 2.2 μm diameters, immersed in agar. The measured attenuation coefficient and speed of sound of the reference phantom were 0.477 dB/MHz/cm and 1540 m/s, respectively. The reference phantom was scanned using the same US system and transducer settings.

As acoustic waves are attenuated as they propagate through the intervening tissue layers, this can affect the backscatter parameter estimation. Attenuation correction was performed using the assumed attenuation coefficients of 2.0 dB/cm/MHz and 0.6 dB/cm/MHz for the skin and the tumour, respectively [24]. Manual segmentation was performed on US B-mode images to identify the tumour ROI and the water-to-skin interface. We assumed a uniform skin thickness of 0.5 mm. The analysis frequency bandwidth is approximately 10 MHz to 25 MHz.

Acoustic scatterer properties that include average scatterer diameters (ASD) and average acoustic concentration (AAC) were obtained by fitting the measured backscatter coefficient (BSC) with a theoretically derived BSC. BSC was calculated using the reference phantom technique. The measured BSC is expressed as

1 $$ {\sigma}_s(f)={\sigma}_r(f)\frac{{\left|{S}_s(f)\right|}^2}{{\left|{S}_r(f)\right|}^2}{e}^{\left\{4\left({\alpha}_s(f)-{\alpha}_r(f)\right)\left(R+\frac{\Delta z}{2}\right)\right\}}, $$

where *S*_*s*_(*f*) and *S*_*r*_(*f*) are the spectra from the sample and the reference phantom, respectively. *α*_*S*_ and *α*_*R*_ are the attenuation coefficients of the sample and the reference phantom, respectively. *R* is the distance from the face of the transducer to the proximal side of the sliding window and ∆*z* is the length of the window. *σ*_*r*_ is the reference BSC. The theoretical BSC is expressed as

2 $$ {\sigma}_{theor}(f)=C{f}^4{a}_{eff}^6\overline{n}{\gamma}_0^2F\left(f,{a}_{eff}\right), $$

where constant *C* = *π*^4^/(36*c*^4^), with *c* is the speed of sound in the medium. *a*_*eff*_ is the effective radius of the scatterers. The product $\overline{n}{\gamma}_0^2$ is the AAC, with $\overline{n}$ is the number of scatterers per unit volume and ${\gamma}_0^2$ is the mean-square variation in the acoustic impedance between a scatterer and the background medium [25]. F(f, a_eff_) is the form-factor that describes changes in the BSC as a function of frequency [25]. Two form-factor models that include the spherical Gaussian model (SGM) and fluid-filled sphere model (FFSM) were evaluated. The estimated scatterer diameter is the one that corresponds to the best-fitted BSC.

### Histopathology

Mice were euthanized at experimental endpoints and all tumour specimens were immediately excised. Tumour samples were dissected into halves, with one half fixed in optical gel subsequently flash-frozen with liquid nitrogen, and the other half fixed in 10% neutral-buffered formalin (Fisher Scientific Canada, Ottawa Ontario, Canada). Formalin-fixed specimens were stored for up to 48 h at room temperature, then were transferred into 70% ethanol solution, and stored at 4 °C for 24 h. In preparation for staining, tumour samples were embedded in paraffin blocks. Specimens were stained with terminal deoxynucleotidyl transferase dUTP nick end labeling (TUNEL) for detection of apoptotic cells, and hematoxylin and eosin (H&E) for identification of tissue and cellular structure. All staining was completed by the Pathology Research Program, University Health Network, Toronto, ON, Canada.

Tissue specimens were digitized and captured using a Leica CD100 microscope (1MPixel Leica DC100 video camera, 2 GHz PC operating Leica IM1000 software) at 20x objective lens.

### Statistical analysis

Statistical analysis was conducted using GraphPad Prism (GraphPad Software, La Jolla, CA, USA) using one-way analysis of variance (ANOVA) followed by Bonferroni’s selected comparisons test. *P-values* (*p* < 0.05) were considered statistically significant and are indicated by asterisk (*).

## Results

### Cell death and tissue structural changes followed ultrasound-stimulated microbubbles and hyperthermia

**Fig. 1:**
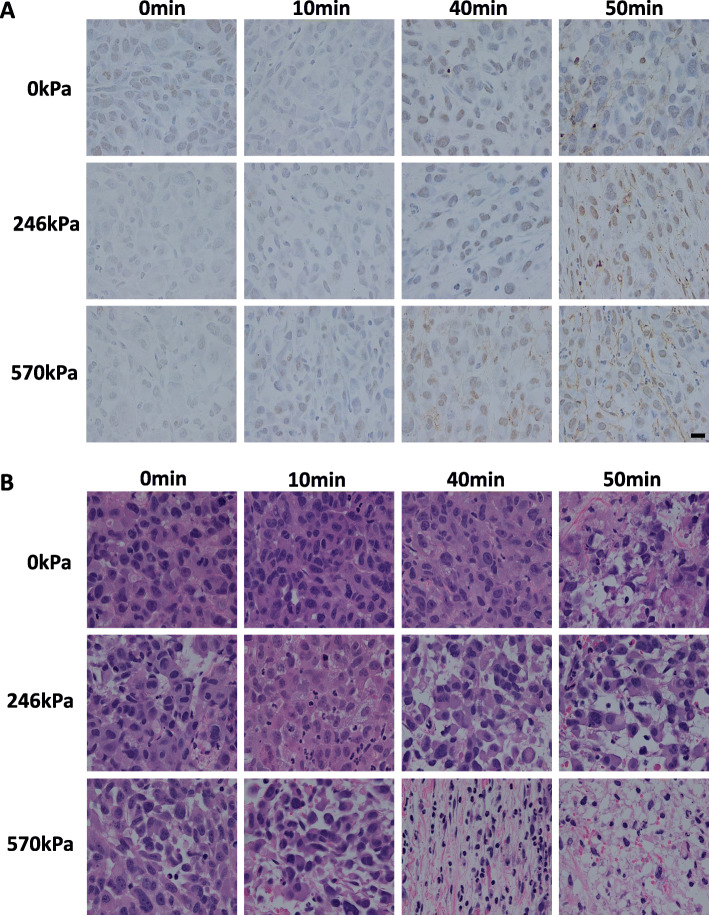
Effects of USMB and HT on tumour cell death. High magnification light microscope images of **A** TUNEL and **B** H&E stained histological sections of PC3 xenografts. **A** Representative images of PC3 xenograft treated with 1% microbubble stimulated at varying US pulses 246 kPa & 570 kPa and varied HT (10, 40, and 50 min). An increase in HT duration combined with USMB demonstrated higher cell death (brown color). Scale bar: 50 μm. **B** H&E-stained PC3 xenografts captured with a high magnification light microscope revealed a significantly higher number of distorted nuclei in the combined USMB and HT treated group compared to the untreated group or USMB alone. Combined treated cells exhibited morphological characteristics of apoptosis as well as necrosis. Scale bar: 50 μm. The image represents a representative experiment (from 3 to 4 independent experiments)

Representative images of tumour sections stained with TUNEL and H&E are displayed in Fig. 1. Cell death was assessed using the TUNEL assay that detects deoxyribonucleic acid (DNA) breakage during the process of apoptosis and H&E staining was used to characterize the tissue sample’s morphology. The top rows of Fig. 1, A and B depicts treatment condition with no US pulses and only HT treatment (0, 10, 40, and 50 min). The middle row depicts tumour section treated with 246 kPa and HT (0, 10, 40, and 50 min) and the bottom row shows stained tumour cross-sections upon exposure of 570 kPa and HT (0, 10, 40, and 50 min). Tissue sections treated with 570 kPa + 40 min and 570 kPa + 50 min treatments revealed a greater area of cell death (brown color). Our result from H&E staining revealed that tissue treated with a combination of USMB and HT demonstrated greater tissue damage with cells exhibiting characteristics of both apoptosis and necrosis.

Earlier studies have demonstrated similar outcomes with cells undergoing apoptosis upon exposure to USMB and radiation. Increasing radiation treatment with a combination of USMB demonstrated higher areas of tumour cell death. Similarly, significant tissue morphological changes were also reported with an increase in erythrocytes area as a result of vascular damage [26].

### Ultrasonic scattering properties of cell death

**Fig. 2:**
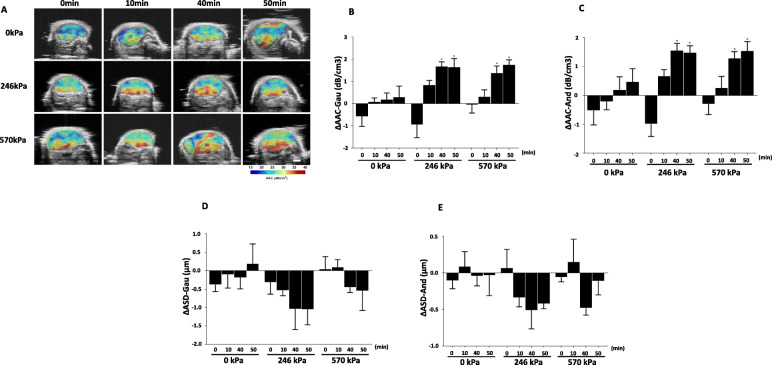
Parametric maps of the AAC and changes in quantitative ultrasound parameters with different treatments. **A** Representative post-treatment (24-h) parametric images of AAC obtained using the SGM, overlaid on B-mode images, **B** Bar plots of averaged changes in AAC from SGM, **C** bar plots of averaged changes in AAC from FFSM, **D** Bar plots of averaged changes of ASD from SGM, and **E** Bar plots of averaged changes in ASD from FFSM from different treatment groups. **A** We observed increases in AAC from the combination of treatment groups: 246 kPa + 40 min, 246 kPa + 50 min, 570 kPa + 40 min, and 570 kPa + 50 min, relative to that of the control group (0 kPa + 0 min). These indicate an increase in acoustic back-scattering that reflects nuclear structure alteration as the tumour cells undergo cell death, as a result of a combination of cancer treatment: USMB-induced vascular disruption and HT. **B** Bar plots of changes in AAC from SGM from all treatment groups. **C** Bar plots of changes in AAC from FFSM from all treatment groups. The height of the bar represents the mean-value from each group. Error bars represent the standard error of the mean. Increases in AAC are apparent, particularly in the combination of treatment groups: 246 kPa + 40 min, 246 kPa + 50 min, and 570 kPa + 50 min. These changes are statistically significant (*p* < 0.05), as indicated by the asterisks, compared to that of the control group (0 kPa + 0 min). We used a one-way ANOVA followed by Bonferroni’s selected comparisons test to investigate for any statistical difference between the treatment groups. **D** Bar plots of changes in ASD from SGM for all treatment groups. **E** Bar plots of changes in ASD from FFSM for all treatment groups. We observed a decreasing trend in the mean-value of ASD as a function of HT duration from the combination of treatment groups. Nonetheless, the observed trend was not statistically significant. In **B**, **C**, **D**, and **E** ΔAAC-SGM, ΔAAC-FFSM, ΔASD-SGM, and ΔASD-FFSM indicates changes in AAC and ASD parameter from the SGM and FFSM models, respectively

Figure 2A shows representative post-treatment (24-h) parametric images of AAC from SGM for all treatment groups. The range of the color bar is from 15 up to 40 dB/cm^3^ for all images. We observed a significant increase in AAC, in comparison with the control group, particularly in the 246 kPa + 40 min, 246 kPa + 50 min, 570 kPa + 40 min, and 570 kPa + 50 min combined treated groups. These changes correlate well with morphological alterations associated with cell death as observed in the H&E and TUNEL images (Fig. 1A & B). AAC serves as a non-invasive imaging biomarker of cell death. Figure 2B presents average changes of AAC from SGM for all treatment conditions. Error bars indicate the standard error of the mean, from the respective group. Significant increases in AAC were observed in four combined treated groups: 246 kPa + 40 min, 246 kPa + 50 min, 570 kPa + 40 min, and 570 kPa + 50 min. These increases were 1.67 ± 0.22 dB/cm^3^, 1.64 ± 0.40 dB/cm^3^, 1.38 ± 0.32 dB/cm^3^, and 1.75 ± 0.23 dB/cm^3^, respectively. Using the FFSM model, we also observed the same trend of increase in AAC as a function of treatment, as shown in Fig. 2C. Increases in AAC were 1.55 ± 0.25 dB/cm^3^, 1.48 ± 0.24 dB/cm^3^, 1.28 ± 0.23 dB/cm^3^, 1.53 ± 0.33 dB/cm^3^ for 246 kPa + 40 min, 246 kPa + 50 min, 570 kPa + 40 min, and 570 kPa + 50 min treatment groups, respectively. Changes in AAC from 246 kPa + 40 min, 246 kPa + 50 min, and 570 kPa + 50 min treatment groups were found to be statistically significantly different (*p* < 0.05) compared to that of the control group (0 kPa + 0 min). Statistical analysis was performed using one-way analysis of variance (ANOVA) with selected comparisons using the Bonferroni method to test for any statistically significant difference between different treatment groups. Increases in AAC were consistent with cell death that was observed in the histological images. Figure 2D & E depicts mean changes in ASD from SGM and ASD from FFSM, respectively, for all treatment groups. For a given US pressure, we observed a decrease in the mean-value of ASD as a function of HT duration. Nonetheless, these changes were not statistically significant (*p* > 0.05). Using FFSM, we also observed the same trend of decreasing ASD as a function of HT duration for a given US pressure. Still, the changes in ASD from FFSM were also not statistically significant.

**Fig. 3:**
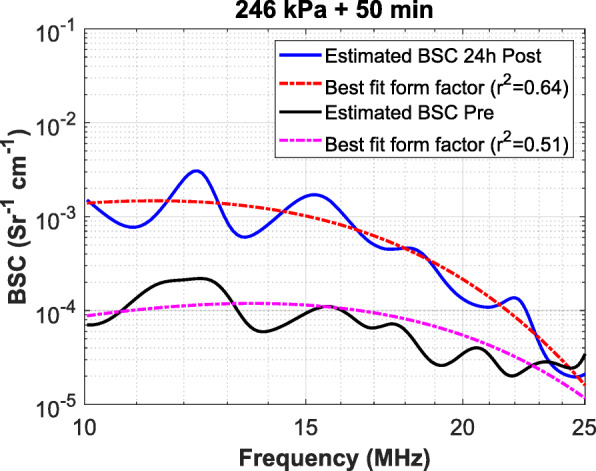
Representative plot of the estimated BSCs pre-and post-treatment, along with their respective best-fit form factor from the SGM model. These are shown from two combination of treatment groups 246 kPa + 50 min (graph depicting plot of the estimated BSCs pre-and post-treatment 570 kPa + 50 min is shown in Supplementary Figure 2). The BSCs are estimated from 50 individual attenuation-corrected normalized power spectra from two-dimensional tumour ROI. An increase in the acoustic backscattering resulting from cell deaths 24-h post-treatment, relative to the pre-treatment, is apparent from the BSC plot

Representative plot of the estimated BSCs, along with their best-fit form factor from SGM for 246 kPa + 50 min treatment group is depicted in Fig. 3. Nuclear structure alteration arising from cell death is reflected in the increase in the BSCs as shown in Fig. 3. An increase in cell death as a result of a combination of treatments results in more acoustic backscattering as quantified using the BSC.

**Fig. 4:**
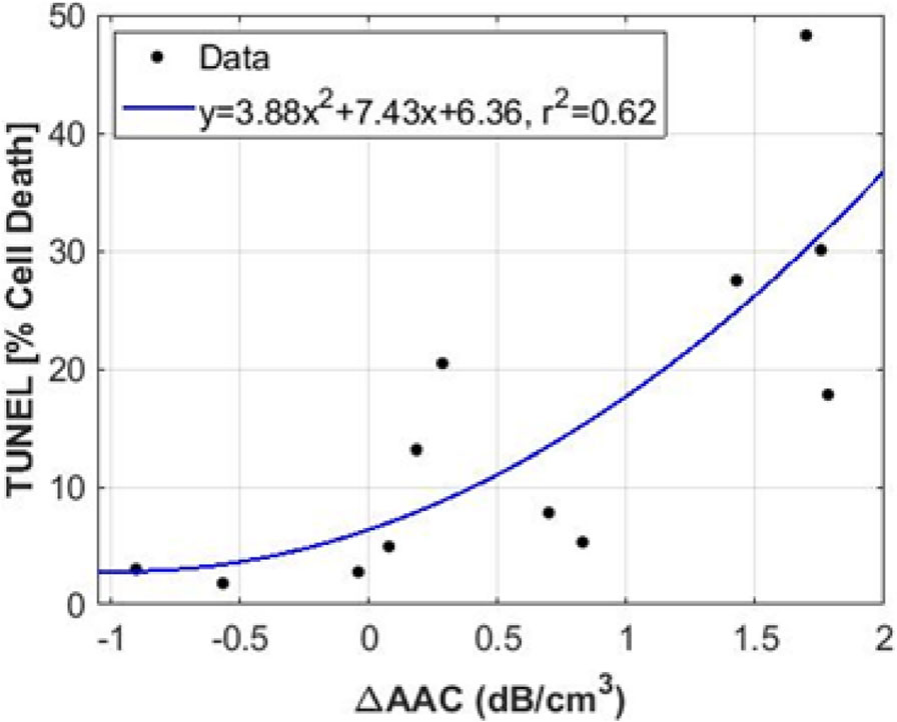
Representative dot plot for AAC and cell death. Correlation between changes in AAC and cell death fraction from TUNEL staining. Scatter plots of cell death fraction versus changes in AAC from all treatment groups. Apoptotic cell death percentile (brown area) was quantified from TUNEL images. We observed an increase in cell death as a function of HT duration for each combination of the treatment group. Increases in AAC due to cell deaths may have been caused by nuclear fragmentation and filling of the extracellular space with cell debris as tumour cells were treated with a combination of treatments of US-mediated vascular disruption and HT. Regression analysis of cell death fraction versus changes in AAC showed a strong correlation (*r*^*2*^ = 0.62). The best quadratic curve-fitting to the data is shown in blue, with the goodness-of-fit function shown in the inset legend

A scatter plot of percent cell deaths versus changes in AAC from all treatment groups is shown in Fig. 4. Apoptotic cell death was quantified from TUNEL images. A strong correlation between changes in AAC and cell deaths was observed (*r*^*2*^ = 0.62). The best quadratic curve-fitting to the data is shown in blue, with the goodness-of-fit shown in the inset legend. Larger changes in AAC imply an increase in cell death.

## Discussion

Previous studies have shown the synergistic effect of USMB-induced vascular disruption and radiation therapy in killing tumour cells [27–29]. Here, we demonstrated a novel combination of cancer treatments in USMB-induced vascular disruption and HT on the animal model of human prostate cancer (PC3). Our results suggest that the combination of USMB and HT induces more cell death compared to either USMB alone or HT alone. Furthermore, we also showed that QUS spectroscopy can be used to non-invasively assess the efficacy of cancer treatment. Acoustic scattering properties obtained from high US frequencies can be used as imaging biomarkers of cell death resulting from cancer treatment. Two scattering properties including ASD and AAC were estimated using SGM and FFSM form factor models to investigate the potential changes in scatterer sizes and concentrations in response to treatment. Histopathology images confirmed a prominent increase in cell death in the combined treatment groups: 246 kPa + 40 min, 246 kPa + 50 min, 570 kPa + 40 min, and 570 kPa + 50 min, in comparison with the control (0 kPa + 0 min). Changes in AAC related to cell death were consistent with changes in the surrogate measures of AAC (mid-band fit (MBF) and 0-MHz intercept (SI)) and changes in backscatter intensity that had been observed previously both from in vitro and in vivo studies [13, 29].

Based on our working model of ultrasonic scattering properties related to cell death, nuclear alterations are predominantly responsible for the increase in AAC. AAC is related to the backscatter amplitude because it is the coefficient of the BSC. Changes in the magnitude and the frequency dependence of ultrasonic backscatter have been shown earlier to correlate with changes in the nuclear structure of the cell [16]. An increase in AAC (and therefore the BSC) can be attributed to cell death as nuclear fragmentation occurring inside the tumour cells and cellular debris filling the extracellular space as tumour cells treated with anticancer treatment. The increase is evident from the BSC plots of pre-and post-treatment for the 246 kPa + 50 min and 570 kPa + 50 min shown in Fig. 3.

The main mechanism of HT is to induce direct cell kill by destructing proteins and the structure within cells and to sensitize cells to radiation and chemotherapy, through the application of extreme heat (> 43 °C) [30–32]. In this work, we explored a combination of USMB + HT as a novel mode of cancer therapeutic. From histology, morphological characteristics of both apoptosis and necrosis as a result of the USMB + HT treatment were observed. We found positive staining of cell death (confirmed by TUNEL and H&E staining), in particular for the combination of treatment groups: 246 kPa + 40 min, 246 kPa + 50 min, 570 kPa + 40 min, and 570 kPa + 50 min compared to the control group (0 kPa + 0 min). In addition, we also observed a decrease in average nuclear size in the combination of treatment groups. This observation is consistent with tumour cells undergoing apoptotic sequence, where they exhibit shrinkage and convolution as typical features of apoptosis. The average nuclear size in the control group was (15.16 ± 1.30 μm) and the minimum average nuclear size of (11.72 ± 0.47 μm) was seen in the 570 kPa + 50 min treatment group. Although a significant decrease in nuclear size was observed (relative to the control), there was no statistically significant difference between groups at different heating times, for a given US pressure (Supplementary Figure 1).

The decrease in nuclear size observed from histology could not be well characterized by scatterer diameters estimates from SGM and FFSM models. Although there was a decrease in ASD as a function of heating duration, the effect was not statistically significant. This can be attributed to the fact that ASD estimates from both models were considerably larger than the cells. The ASD estimates were approximately 40–50 μm. We hypothesize that acoustic scattering arises from larger scattering structures such as clusters of apoptotic cells, nuclear coalescence, and condensation in the proximity of dying tumour cells, along with a contribution from micro-vessels, and not from individual cells. According to the theoretical framework of ultrasonic spectrum analysis developed by Lizzi et al. [33], an increase in the scatterer’s diameters may also cause an increase in the spectral intercept from the linear regression analysis. Although the ASD parameter was observed to decrease, mechanical characteristics such as particle density and concentration of scatterers may have contributed to the increase in acoustic backscattering as quantified through the AAC parameter. In separate studies, Vlad et al. and Franceschini et al. identified the increase in backscattering to arise from an increase in cellular variance as tumour cells undergoing cell death [22, 34]. Here, cellular size variance and micro-vessels might have contributed to the increase in AAC. Variation in scatterer sizes and distributions can be observed qualitatively from representative histological images.

In conclusion, we demonstrated the usefulness of QUS-based biomarkers to noninvasively assess the efficacy of novel anticancer treatment. HT was used as the primary cancer treatment, in conjunction with USMB-induced vascular disruption to treat prostate tumour xenograft. Furthermore, scatterer property-based biomarkers from QUS spectroscopy analysis were used as non-invasive imaging biomarkers of cell death. In particular, the changes in AAC showed a good correlation with cell death fraction. QUS spectroscopy is a potential imaging method for monitoring response to anticancer treatment.

## Supplementary Information

**Additional file 1: Figure S1.** Nuclear size estimation of PC3 xenograft sections. Nuclear size assessment in PC3 tumour cross-sections following 24 h of treatment. Combined treatment of USMB and HT (246 kPa + 0 min; 246 kPa + 10 min; 246 kPa + 40 min; 246 kPa + 50 min; 570 kPa + 0 min; 570 kPa + 10 min; 570 kPa + 40 min and 570 kPa + 50 min) resulted in a significant difference in nuclear size compared to control (0 kPa + 0 min). An asterisk indicates *P*-values (*p* < 0.05) (*).

**Additional file 2: Figure S2.** BSCs plot obtained from an in vivo prostate tumour model pre-and-post 24 h after USMB and HT treatment. A representative plot of the measured BSC and its best-fit theoretical BSC from the SGM model for 570 kPa + 50 min HT group at pre-and post-treatment is shown. An increase in backscattering is observed at 24 h post-treatment.

## Data Availability

All datasets included in this study/manuscript and available from the corresponding author upon request.

## References

[1] Anderson JB, Webb AJ (1987). Fine-needle aspiration biopsy and the diagnosis of thyroid cancer. Br J Surg.

[2] Krücker J, Xu S, Glossop N, Viswanathan A, Borgert J, Schulz H, Wood BJ (2007). Electromagnetic tracking for thermal ablation and biopsy guidance: clinical evaluation of spatial accuracy. J Vasc Interv Radiol.

[3] Hiraki T, Mimura H, Gobara H, Iguchi T, Fujiwara H, Sakurai J, Matsui Y, Inoue D, Toyooka S, Sano Y, Kanazawa S (2009). CT fluoroscopy-guided biopsy of 1,000 pulmonary lesions performed with 20-gauge coaxial cutting needles: diagnostic yield and risk factors for diagnostic failure. Chest..

[4] Natarajan S, Marks LS, Margolis DJA, Huang J, Macairan ML, Lieu P (2011). Clinical application of a 3D ultrasound-guided prostate biopsy system. Urol Oncol.

[5] Pinto PA, Chung PH, Rastinehad AR, Baccala AA, Kruecker J, Benjamin CJ (2011). Magnetic resonance imaging/ultrasound fusion guided prostate biopsy improves cancer detection following transrectal ultrasound biopsy and correlates with multiparametric magnetic resonance imaging. J Urol.

[6] Czarnota GJ, Kolios MC, Vaziri H, Benchimol S, Ottensmeyer FP, Sherar MD, Hunt JW (1997). Ultrasonic biomicroscopy of viable, dead and apoptotic cells. Ultrasound Med Biol.

[7] Czarnota GJ, Kolios MC, Abraham J, Portnoy M, Ottensmeyer FP, Hunt JW, Sherar MD (1999). Ultrasound imaging of apoptosis: high-resolution non-invasive monitoring of programmed cell death in vitro, in situ and in vivo. Br J Cancer.

[8] Oelze ML, Mamou J (2016). Review of quantitative ultrasound: envelope statistics and backscatter coefficient imaging and contributions to diagnostic ultrasound. IEEE Trans Ultrason Ferroelectr Freq Control.

[9] Feleppa EJ, Mamou J, Porter CR, MacHi J (2011). Quantitative ultrasound in cancer imaging. Semin Oncol.

[10] Lizzi FL, Ostromogilsky M, Feleppa EJ, Rorke MC, Yaremko MM (1987). Relationship of ultrasonic spectral parameters to features of tissue microstructure. IEEE Trans Ultrason Ferroelectr Freq Control.

[11] Lizzi FL, Astor M, Feleppa EJ, Shao M, Kalisz A (1997). Statistical framework for ultrasonic spectral parameter imaging. Ultrasound Med Biol.

[12] Vlad RM, Alajez NM, Giles A, Kolios MC, Czarnota GJ (2008). Quantitative ultrasound characterization of cancer radiotherapy effects in vitro. Int J Radiat Oncol Biol Phys.

[13] Banihashemi B, Vlad R, Debeljevic B, Giles A, Kolios MC, Czarnota GJ (2008). Ultrasound imaging of apoptosis in tumor response: novel preclinical monitoring of photodynamic therapy effects. Cancer Res.

[14] Sadeghi-Naini A, Papanicolau N, Falou O, Tadayyon H, Lee J, Zubovits J, Sadeghian A, Karshafian R, al-Mahrouki A, Giles A, Kolios MC, Czarnota GJ (2013). Low-frequency quantitative ultrasound imaging of cell death in vivo. Med Phys.

[15] Sannachi L, Tadayyon H, Sadeghi-Naini A, Tran W, Gandhi S, Wright F (2015). Non-invasive evaluation of breast cancer response to chemotherapy using quantitative ultrasonic backscatter parameters. Med Image Anal.

[16] Tadayyon H, Sannachi L, Sadeghi-Naini A, Al-Mahrouki A, Tran WT, Kolios MC (2015). Quantification of ultrasonic scattering properties of in vivo tumor cell death in mouse models of breast cancer. Transl Oncol.

[17] Kim HC, Al-Mahrouki A, Gorjizadeh A, Sadeghi-Naini A, Karshafian R, Czarnota GJ (2014). Quantitative ultrasound characterization of tumor cell death: ultrasound-stimulated microbubbles for radiation enhancement. Chen X, editor. PLoS One.

[18] Kim HC, Al-Mahrouki A, Gorjizadeh A, Karshafian R, Czarnota GJ (2013). Effects of biophysical parameters in enhancing radiation responses of prostate tumors with ultrasound-stimulated microbubbles. Ultrasound Med Biol.

[19] Sharma D, Giles A, Hashim A, Yip J, Ji Y, Do NNA (2019). Ultrasound microbubble potentiated enhancement of hyperthermia-effect in tumours. Lebedeva I V., editor. PLoS One.

[20] Sharma D, Cartar H, Law N, Giles A, Farhat G, Oelze M (2020). Optimization of microbubble enhancement of hyperthermia for cancer therapy in an in vivo breast tumour model. PLoS One.

[21] Sun C, Pye SD, Browne JE, Janeczko A, Ellis B, Butler MB (2012). The speed of sound and attenuation of an IEC agar-based tissue-mimicking material for high frequency ultrasound applications. Ultrasound Med Biol.

[22] Franceschini E, Balasse L, Roffino S, Guillet B (2019). Probing the cellular size distribution in cell samples undergoing cell death. Ultrasound Med Biol.

[23] Yao LX, Zagzebski JA, Madsen EL. Backscatter Coefficient Measurements Using a Reference Phantom to Extract Depth-Dependent Instrumentation Factors. Ultrasonic Imaging. 1990;12(1):58-70. 10.1177/016173469001200105. 10.1177/016173469001200105 2184569

[24] Vlad RM, Brand S, Giles A, Kolios MC, Czarnota GJ (2009). Quantitative ultrasound characterization of responses to radiotherapy in cancer mouse models. Clin Cancer Res.

[25] Insana MF, Hall TJ (1990). Parametric ultrasound imaging from backscatter coefficient measurements: image formation and interpretation. Ultrason Imaging.

[26] Tran WT, Sannachi L, Papanicolau N, Tadayyon H, Al Mahrouki A, El Kaffas A (2016). Quantitative ultrasound imaging of therapy response in bladder cancer in vivo. Oncoscience..

[27] El Kaffas A, Gangeh MJ, Farhat G, Tran WT, Hashim A, Giles A (2018). Tumour vascular shutdown and cell death following ultrasound-microbubble enhanced radiation therapy. Theranostics..

[28] Czarnota GJ, Karshafian R, Burns PN, Wong S, Al Mahrouki A, Lee JW (2012). Tumor radiation response enhancement by acoustical stimulation of the vasculature. Proc Natl Acad Sci U S A.

[29] Lee J, Karshafian R, Papanicolau N, Giles A, Kolios MC, Czarnota GJ (2012). Quantitative ultrasound for the monitoring of novel microbubble and ultrasound radiosensitization. Ultrasound Med Biol.

[30] Wust P, Hildebrandt B, Sreenivasa G, Rau B, Gellermann J, Riess H (2002). Hyperthermia in combined treatment of cancer. Lancet Oncol.

[31] Steeves RA (1992). Hyperthermia in cancer therapy: where are we today and where are we going?. Bull N Y Acad Med.

[32] Jha S, Sharma PK, Malviya R (2016). Hyperthermia: role and risk factor for cancer treatment. Achiev Life Sci.

[33] Coleman J, Greenebaum M, Feleppa EJ, Elbaum M (1983). Theoretical framework for spectrum analysis in ultrasonic tissue characterization. J Acoust Soc Am.

[34] Vlad RM, Saha RK, Alajez NM, Ranieri S, Czarnota GJ, Kolios MC (2010). An increase in cellular size variance contributes to the increase in ultrasound backscatter during cell death. Ultrasound Med Biol.

