# Supplementary material for: In vivo assessment of prostate cancer response using quantitative ultrasound characterization of ultrasonic scattering properties
Source: BMC Cancer. 2021 Sep 3;21:991. doi: 10.1186/s12885-021-08706-7 (PMC8417963; doi:10.1186/s12885-021-08706-7)
Supplement: Supplementary file 1 — Additional file 1: Figure S1. Nuclear size estimation of PC3 xenograft sections. Nuclear size assessment in PC3 tumour cross-sections following 24 h of treatment. Combined treatment of USMB and HT (246 kPa + 0 min; 246 kPa + 10 min; 246 kPa + 40 min; 246 kPa + 50 min; 570 kPa + 0 min; 570 kPa + 10 min; 570 kPa + 40 min and 570 kPa + 50 min) resulted in a significant difference in nuclear size compared to control (0 kPa + 0 min). An asterisk indicates P-values (p < 0.05) (*). [file 12885_2021_8706_MOESM1_ESM.docx]

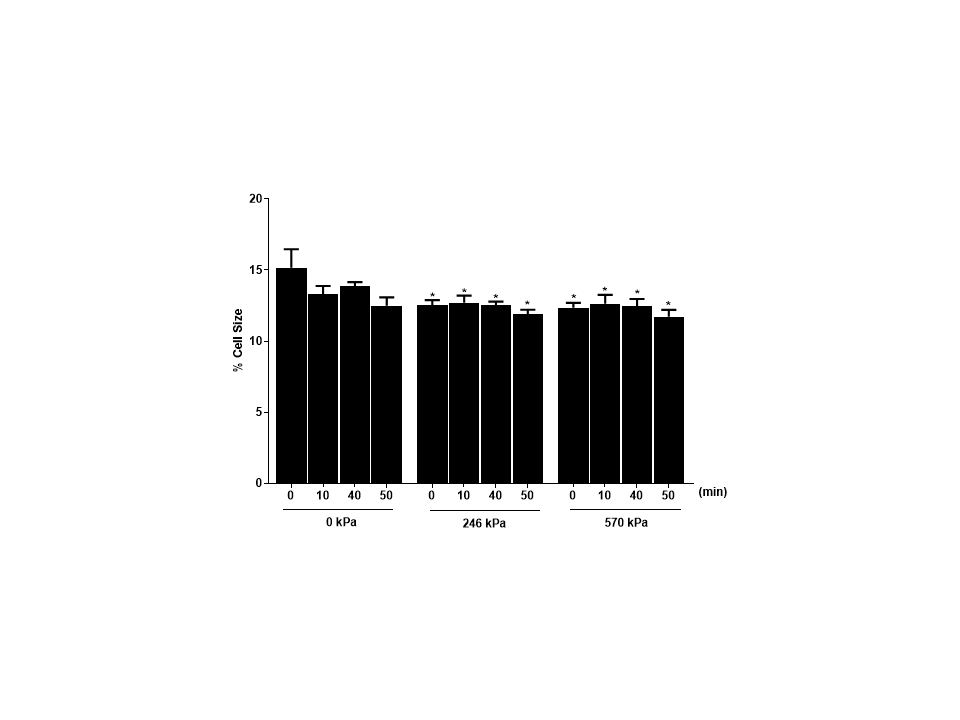
Supplementary Figure 1

**Figure S1. Nuclear size estimation of PC3 xenograft sections.**Nuclear size assessment in PC3 tumour cross-sections following 24h of treatment. Combined treatment of USMB and HT (246 kPa + 0 min; 246 kPa + 10 min; 246 kPa + 40 min; 246 kPa + 50 min; 570 kPa + 0 min; 570 kPa + 10 min; 570 kPa + 40 min and 570 kPa + 50 min) resulted in a significant difference in nuclear size compared to control (0 kPa + 0 min). An asterisk indicates P-values (p < 0.05) (*).
