# Supplementary material for: In vivo assessment of prostate cancer response using quantitative ultrasound characterization of ultrasonic scattering properties
Source: BMC Cancer. 2021 Sep 3;21:991. doi: 10.1186/s12885-021-08706-7 (PMC8417963; doi:10.1186/s12885-021-08706-7)
Supplement: Supplementary file 2 — Additional file 2: Figure S2. BSCs plot obtained from an in vivo prostate tumour model pre-and-post 24 h after USMB and HT treatment. A representative plot of the measured BSC and its best-fit theoretical BSC from the SGM model for 570 kPa + 50 min HT group at pre-and post-treatment is shown. An increase in backscattering is observed at 24 h post-treatment. [file 12885_2021_8706_MOESM2_ESM.docx]

Supplementary Figure 2

**
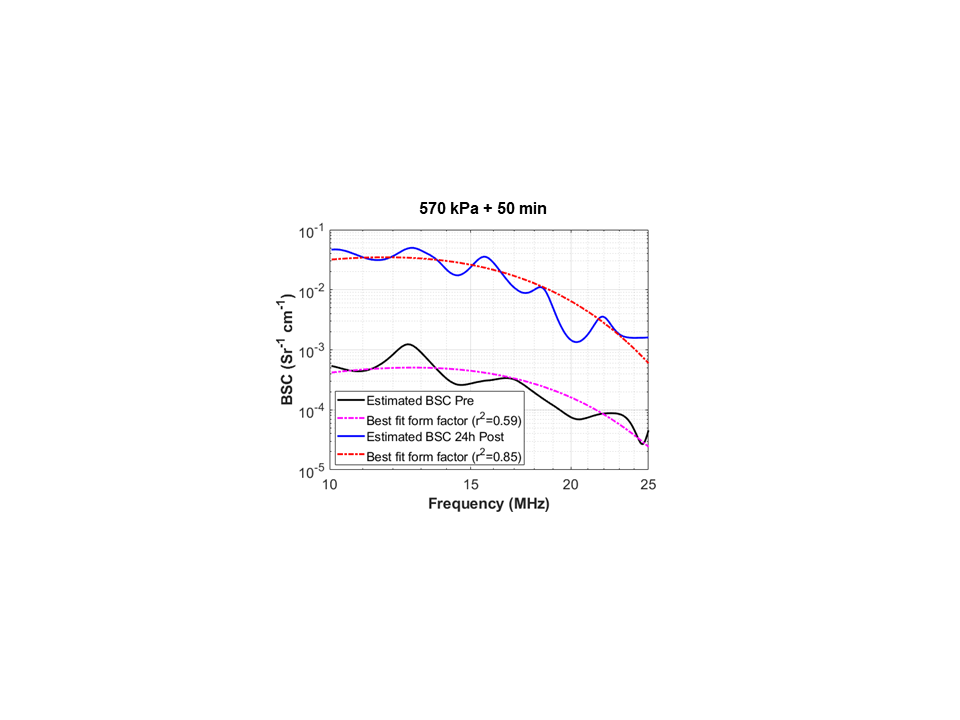
**

**Figure S2. BSCs plot obtained from an *in vivo* prostate tumour model pre-and-post 24 hours after USMB and HT treatment.**A representative plot of the measured BSC and its best-fit theoretical BSC from the SGM model for 570 kPa + 50 min HT group at pre-and post-treatment is shown. An increase in backscattering is observed at 24 hours post-treatment.
